# Supplementary figures and images for: Time-course changes in mental distress and their predictors in response to the coronavirus disease 2019 (COVID-19) pandemic: A longitudinal multi-site study of hospital staff
Source: PLoS One. 2023 Oct 5;18(10):e0292302. doi: 10.1371/journal.pone.0292302 (PMC10553228; doi:10.1371/journal.pone.0292302)

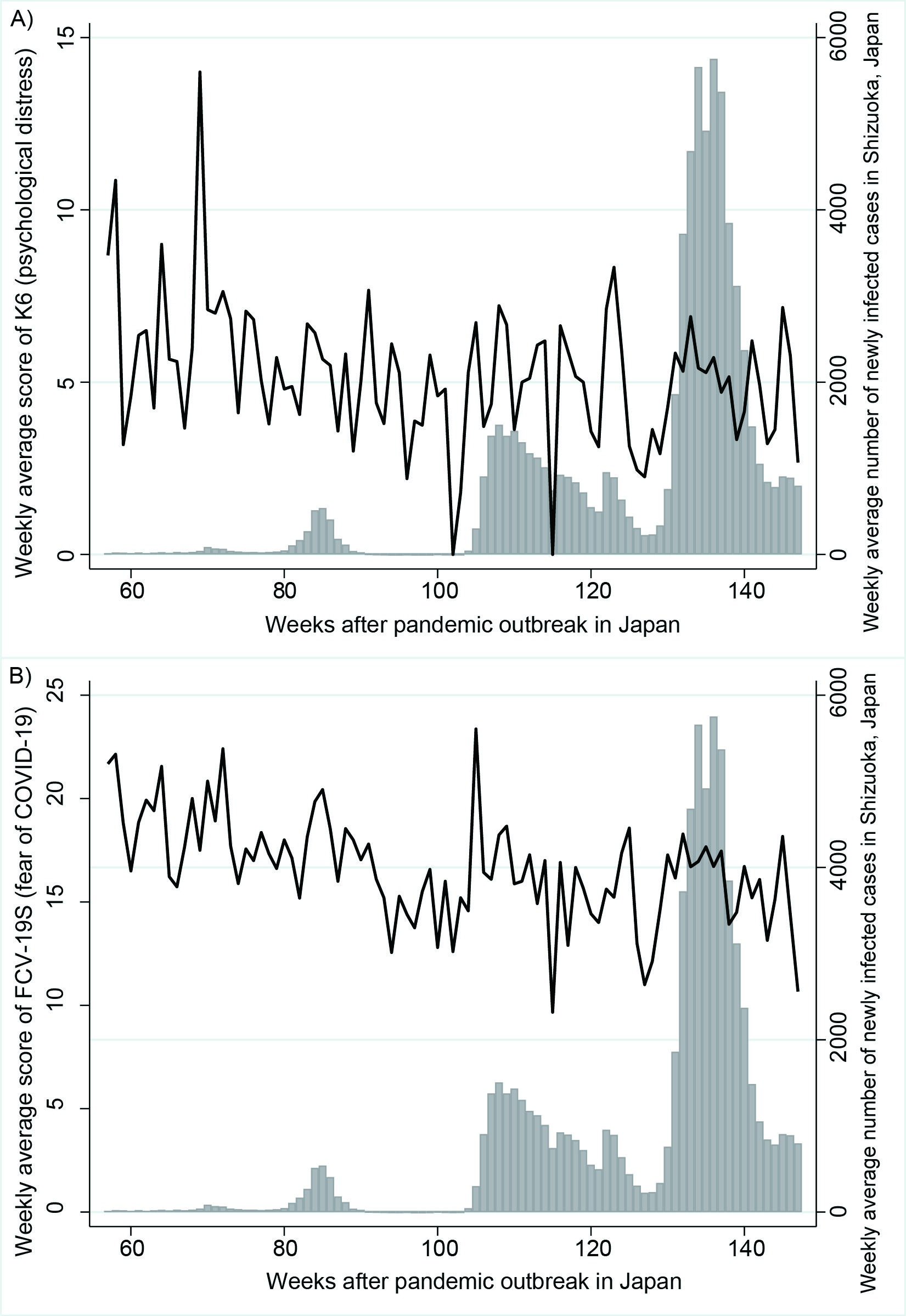

Supplement: S1 Fig — Weekly average number of newly infected COVID-19 cases and K6 (A) or FCV-19S (B) scores were shown during the observation period. (TIF) [file pone.0292302.s001.tif]
