# Supplementary material for: Time-course changes in mental distress and their predictors in response to the coronavirus disease 2019 (COVID-19) pandemic: A longitudinal multi-site study of hospital staff
Source: PLoS One. 2023 Oct 5;18(10):e0292302. doi: 10.1371/journal.pone.0292302 (PMC10553228; doi:10.1371/journal.pone.0292302)
Supplement: S1 Table — †The day of the COVID-19 outbreak in Japan is defined as “week 1” (January 16, 2020). *Statistical significance after Bonferroni correction (p < 0.0167). Abbreviations: B, regression coefficient; 95% CI, 95% confidence interval. (DOCX) [file pone.0292302.s002.docx]

**S1 Table. Effects of time and trait anxiety or occupation type on K6 scores for 100 weeks or earlier and after 100 weeks**

|  | 100 weeks or earlier | | After 100 weeks | |
| --- | --- | --- | --- | --- |
|  | Model 1 | Model 2 | Model 1 | Model 2 |
|  | B (95% CI) | B (95% CI) | B (95% CI) | B (95% CI) |
| Time (month) | -.04 (-.06, -.01)* | .02 (-.09, .14) | -.01 (-.04, .03) | .04 (-.09, .16) |
| Trait anxiety (below average group is reference) | | | | |
| High | .65 (.19, 1.12)* | 37.5 (-38.8, 113.8) | .97 (.45, 1.49)* | 138.3 (43.5, 233.0)* |
| Very high | 1.31 (.86, 1.77)* | 71.8 (-3.38, 146.9) | 1.67 (1.15, 2.20)* | 118.5 (25.4, 211.5)* |
| Time × Trait anxiety | | | | |
| High |  | -.05 (-.15, .05) |  | -.18 (-.31, -.06)* |
| Very high |  | -.10 (-.20, .01) |  | -.16 (-.28, -.03) -109.5* |
| Occupation type (other hospital staff is reference) | | | | |
| Doctor | -.72 (-1.45, .01) | -1.83 (-9.47, 86.8) | .01 (-.78, .80) | -68.4 (-162.3, 25.6) |
| Nurse | -.12 (-.47, .23) | -7.41 (-61.9, 47.0) | -.25 (-.72, .22) | -109.5 (-174.1, -45.0)* |
| Time × Occupation type | | | | |
| Doctor |  | .001 (-.12, .12) |  | .09 (-.03, .22) |
| Nurse |  | .01 (-.06, .08) |  | .15 (.06, .23)* |
| Background characteristics | | | | |
| Age | .002 (-.01, .01) | .00 (-.01, .01) | -.002 (-.02, .02) | .002 (-.02, .02) |
| Gender (female) | .24 (-.18, .66) | .26 (-.15, .67) | .74 (.14, 1.34)* | .81 (.21, 1.41)* |
