# Supplementary material for: Time-course changes in mental distress and their predictors in response to the coronavirus disease 2019 (COVID-19) pandemic: A longitudinal multi-site study of hospital staff
Source: PLoS One. 2023 Oct 5;18(10):e0292302. doi: 10.1371/journal.pone.0292302 (PMC10553228; doi:10.1371/journal.pone.0292302)
Supplement: S2 Table — †The day of the COVID-19 outbreak in Japan is defined as “week 1” (January 16, 2020). *Statistical significance after Bonferroni correction (p < 0.0167). Abbreviations: B, regression coefficient; 95% CI, 95% confidence interval. (DOCX) [file pone.0292302.s003.docx]

**S2 Table. Effects of time and trait anxiety or occupation type on FCV-19S scores for 100 weeks or earlier and after 100 weeks**

|  | 100 weeks or earlier | | After 100 weeks | |
| --- | --- | --- | --- | --- |
|  | Model 1 | Model 2 | Model 1 | Model 2 |
|  | B (95% CI) | B (95% CI) | B (95% CI) | B (95% CI) |
| Time (month) | -.38 (-.53, -.23)* | -.50 (-1.05, .04) | -.14 (-.30, .03) | -.35 (-.84, .13) |
| Trait anxiety (below average group is reference) | | | | |
| High | 2.07 (-.10, 4.24) | 189.4 (-135.9, 514.7) | 1.74 (-.57, 4.04) | -230.2 (-606.9, 146.4) |
| Very high | 4.25 (2.10, 6.41)* | 366.1 (29.8, 702.4)* | 3.55 (1.15, 5.95)* | -229.0 (-607.2, 149.2) |
| Time×Trait anxiety | | | | |
| High |  | -.25 (-.69, .19) |  | .31 (-.19, .81) |
| Very high |  | -.49 (-.95, -.03)* |  | .31 (-.19, .82) |
| Occupation type (other hospital staff is reference) | | | | |
| Doctor | -6.82 (-10.3, -3.33)* | -418.1 (-862.5, 26.2) | -1.09 (-4.66, 2.49) | 151.9 (-248.4, 552.2) |
| Nurse | -2.74 (-4.52, -.97)* | -360.3 (-681.3, -39.2)* | -1.27 (-3.55, 1.00) | 7.75 (-300.6, 316.1) |
| Time×Occupation type | | | | |
| Doctor |  | .56 (-.04, 1.16) |  | -.20 (-.74, .33) |
| Nurse |  | .48 (.05, .92)* |  | -.01 (-.42, .40) |
| Background characteristics | | | | |
| Age | -.02 (-.08, .05) | -.01 (-.08, .05) | -.06 (-.15, .02) | -.07 (-.17, .02) |
| Gender (female) | 3.28 (1.17, 5.39)* | 3.56 (1.44, 5.69)* | 2.39 (-.31, 5.09) | 2.25 (-.47, 4.97) |
