# Supplementary material for: Time-course changes in mental distress and their predictors in response to the coronavirus disease 2019 (COVID-19) pandemic: A longitudinal multi-site study of hospital staff
Source: PLoS One. 2023 Oct 5;18(10):e0292302. doi: 10.1371/journal.pone.0292302 (PMC10553228; doi:10.1371/journal.pone.0292302)
Supplement: Attachment — Submitted filename: PONE-D-23-03982R_response.docx [file pone.0292302.s006.docx]

1^st^ June 2023

Dr. Emily Chenette, PhD, and Dr. George Vousden, PhD

Editor-in-Chief, *PLOS ONE*

Dear Dr. Chenette and Dr. Vousden,

Thank you for your kind letter on 5th April 2023, regarding our manuscript PONE-D-23-03982 entitled “**Time-course changes in mental distress and their predictors in response to the coronavirus disease 2019 (COVID-19) pandemic: A long-term longitudinal multi-site study of hospital staff.**” We greatly appreciate the comments and suggestions provided by the editor and reviewers for the revision of our manuscript.

We have substantially revised our manuscript in accordance with your suggestions, as well as those of the reviewers. Our point-by-point responses to all of the comments are presented below; reviewer comments are in italics with our responses immediately following each comment. The revised sections of the text are underlined in each response as well as in the revised manuscript itself.

*Reviewer: 1*

*PLOS One manuscript PONE-D-23-03982, entitled "Time-course changes in mental distress and their predictors in response to the coronavirus disease 2019 (COVID-19) pandemic: A long-term longitudinal multi-site study of hospital staff", is an empirical research report (N = 545 staff members at 18 hospitals treating COVID-19 patients in Shizuoka Prefecture, Japan), of individual differences in the longitudinal trajectory of mental distress over 21 months of COVID-19 waves in Japan. Psychological distress and fear of COVID-19 were initially elevated but then decreased over the course of the investigation, especially for those high in trait anxiety and for specific occupations. The time course of observed effects was also examined with cross-correlation analyses.*

*This is a well-written manuscript on a topic of substantial interest to the readership of PLOS One. The data are quite valuable and were capably analyzed. The results were somewhat countER-intuitive, but the authors have done a good job explaining them.*

*The Introduction appropriately motivates the study and embeds the work in a scholarly and contemporary review of the relevant literature. The authors' remarks about temporal resolution were especially useful.*

We appreciate the reviewer’s kind and careful review.

*1) The psychometric properties of the primary study instruments, K6 and FCV-19S, are not reported.*

We appreciate the reviewer giving us the opportunity to clarify this point. As suggested, the following sentences were added in the revised manuscript:

Methods:

Lines 115-116 of the revised manuscript: “Cronbach’s α of the Japanese version of K6 scale was 0.85 [16].”

Line 120 of the revised manuscript: “Cronbach’s α of the Japanese version of FCV-19S scale was 0.82 [18].”

*2) As the authors point out, the homogeneity of the sample is well suited to exploring individual differences. However, it works against learning anything about contextual factors, such as governmental stringency in response to the pandemic and geographic factors. This is not a defect in the study itself, but it is a constraint on the scope of the questions that might be asked about this topic. The Discussion might address this design feature.*

We thank the reviewer for providing us the opportunity to explore this important issue, and have added both a description of governmental stringency and a brief discussion of this issue in the limitations section as follows:

Methods:

Lines 86 to 92 of the revised manuscript: “Although a state of emergency was declared nationwide, a full lockdown was not legally enforced in Japan. The government of Shizuoka Prefecture asked citizens to avoid both non-essential outings and travel to another prefecture, and requested that they take basic precautionary measures (washing hands, using masks effectively, avoiding closed spaces with poor ventilation, avoiding crowded places with many people nearby, and avoiding close-contact settings, e.g., close-range conversations within 2m). These recommendations did not change significantly during the survey period. Many citizens followed these recommendations.”

Discussion:

Lines 398 to 403 of the revised manuscript: “Third, although the homogeneity of the current study sample is well-suited to exploring individual differences, it could work against learning anything about contextual factors, such as governmental stringency in response to the pandemic or geographic factors. Given that governments worldwide declared different levels of pandemic restrictions, this study was limited in that pandemic conditions in Japan could not be directly compared to those in other nations.”

*3) For the cross-correlation analyses the authors created weekly averages. First of all, were these moving averages? Second, if the causal relationship between infections and distress / fear operates over an interval shorter than a week, won't the effect be obscured by the weekly averaging? This was a bit of a surprise in an article that seemed especially attuned to temporal resolution issues.*

*If the binning of observations was to make "lags" meaningful and comparable, the authors should say so.*

Thank you for giving us the opportunity to clarify this point. For the cross-correlation analyses, we created weekly averages instead of moving averages. The primary reason for creating weekly averages was that the mental health surveys were not completed by each participant on each day. When the unit of time was set to one day, the number of days without surveys, i.e., missing surveys, was large. Therefore, we binned the observations on a weekly basis. As the reviewer pointed out, this reduced temporal resolution and can be considered a limitation of this study. We clarified the reason why the weekly average was employed in the methods section, and added a brief discussion in the limitations section as follows:

Methods:

Lines 184-186 of the revised manuscript: “We utilized weekly averages in the analyses, because mental health surveys were not completed by each participant on each day; therefore, when the unit of time was set to one day, the amount of missing data was very large.”

Discussion:

Lines 391-398 of the revised manuscript: “Second, the lower consistency in the number and interval of data collection periods limited the robustness of findings. To reduce the potential confounding effect, weekly averages were calculated to summarize the observations. These weekly averages could make the temporal resolution insufficient, as the lag between the peak of increase in the number of infections and the peaks of psychological distress and fear of COVID-19 could have occurred at intervals shorter than one week. However, the employed generalized linear mixed model allows for different measurement times and intervals for each individual, and detrended cross-correlation analysis is considered to be less influenced by these limitations.”

In the design of the current study, as we additionally and exploratory aimed to make the lags meaningful, the following revisions were made in the introduction:

Line 62 of the revised manuscript: “Furthermore, high temporal resolution is necessary to test the relationships between mental distress…”

Lines 74-76 of the revised manuscript: “Additionally, using a high temporal resolution analysis of long-term longitudinal data, the time-course changes in the relationship between the pandemic waves and mental distress were also examined.”

*Similarly, the division of the observation period into only two periods and the use of the somewhat arbitrary 100 week cut-off for this division both seem rather coarse from a measurement perspective (vs., perhaps, a cubic or other polynomial model, with anxiety as a continuous moderator). Also, re-running models within categories of interest (e.g., very high versus high anxiety or doctors versus nurses or >100 weeks versus <=100 weeks) and comparing significance levels is not the same as testing interactions, which could be done more directly (e.g., Cohen, Cohen, West, & Aiken, 2003, 3rd ed.).*

Thank you for this insight. As the reviewer pointed out, the division of the observation period into two periods and the use of a 100-week cut-off were somewhat arbitrary. This division was employed because a) the number of newly infected cases differed considerably and b) policies for infection control changed between the first and second half of the observation period, which could have affected mental health differently. In response to this comment, we conducted further analyses on the impact over the entire study period. In order to examine different trends in the number of newly infected cases, psychological distress, and fear of COVID-19 over the entire period, we conducted a detrended cross-correlation analysis (DCCA) using standardized values for the number of newly infected cases as well as K6 and FCV-19S scores. The altered method and the subsequent results are described in the text as follows:

Abstract:

Lines 33-35 of the revised manuscript: “Cross-correlation analyses demonstrated that K6 and FCV-19S exhibited time-course changes, with increases in these indices occurring simultaneously with an increase in infections and decreases delayed by 7 weeks after an increase in infections.”

Methods:

Lines 186-189 of the revised manuscript: “Furthermore, because the number of newly infected cases tended to increase, and because psychological distress and fear of COVID-19 could also have a trend through the period, we conducted a detrended cross-correlation analysis (DCCA) using standardized values.”

Lines 195-196 of the revised manuscript: “GLMM was conducted using STATA version 17.0 [24] and DCCA was conducted using Python version 3.6.”

Results:

Lines 287-290 of the revised manuscript: “The cross-correlation values were positive at lag 0 (r = .25) and negative at lag 7 (r = −.15), indicating that the K6 score tended to increase immediately after an increase in the number of infections and decreased after 7 weeks, although the magnitude of the correlation was relatively small.”

Lines 298-300 of the revised manuscript: “A moderate positive correlation (r = .37) was observed immediately after an increase in the number of infections (lag 0). The value of the correlation then decreased gradually over lag 10 (r = −.13).”

Furthermore, for the results in Table 2 and Table 3, it was not appropriate to repeat the tests for the entire period and for the divided period. We presented only the results for the entire period in the main text of the revised manuscript. However, for the reasons described above, we thought additional analyses would be useful to examine differences in the effect of the number of infections on mental health in the first and second halves of the period, so we have presented the results of a period-divided analysis in the supplemental material of the revised manuscript. In addition, following a suggestion from another reviewer, we changed the analytical method from a linear mixed model to a generalized linear mixed model (GLMM) and corrected multiple comparisons using Bonferroni's method. The revised portions of the methods and results sections are as follows:

Methods:

Lines 190-194 of the revised manuscript: “Additionally, the observation period was divided into two periods (before and after 100 weeks), and a GLMM and DCCA were then conducted for each period. This division was employed because a) the number of newly infected cases differed considerably and b) policies for infection control changed between the first and second half of the observation period, which could have affected mental health differently.”

Results:

Lines 303-308 of the revised manuscript: “In the additional analyses, the cross-correlation between the average number of infections and K6 scores was small during the 100 weeks or earlier period, but after 100 weeks, there was a positive correlation at lag = 0 (r = .27) and a negative correlation at lag = 7 (r = −.32) (S2 Fig). For the cross-correlation between the average number of infections and FCV-19S scores, a similar trend was observed in the early and later periods, with a moderate correlation at lag = 0 (r = .44 and .36, respectively) and decreasing values over lag = 10 (r = −.22 and −.22, respectively) (S3 Fig).”

*4) Line 263, the lag 0 cross-correlation should be a POSITIVE +.27.*

We thank the reviewer for catching this important detail. However, we further conducted analyses on the effect over the entire study period. As a result, the value of cross-correlation at lag 0 was r = .37, which is stated in the revised manuscript as well as in Comment 2 of Reviewer 2.

*5) A *partial* cross-correlation function may expose the effect the authors are seeking more clearly. The apparent smooth periodicity of the CCF may simply be the effects of autocorrelation. The PACF can control for this, leaving only the spikes in effects across lags.*

We appreciate the reviewer’s insight. As suggested, we have tried to apply a partial autocorrelation function (PACF). The following figures show the partial autocorrelation for K6 scores, FCV-19 scores, and the number of newly infected cases, respectively. For the number of newly infected cases, a negative peak was observed at lag 2 week. No clear peak was found for the K6 and FCV-19 scores.

*6. Line 314: Would the authors care to elaborate, even briefly on what they mean by, "The present results further support the possibility that increasing knowledge and access to medical information about infectious diseases can reduce mental distress in response to a pandemic."*

We thank the reviewer for giving us the opportunity to clarify this point. In response to the comment, we have added the following sentences:

Discussion:

Lines 357-362 of the revised manuscript: “Doctors and nurses experienced less psychological distress and fear of COVID-19 than individuals in other hospital occupations. This could be attributed to their comparably easier access to medical information about COVID-19 or their greater ability to take precautions against infection than other hospital workers. Therefore, the present results support the possibility that increasing knowledge and access to medical information about infectious diseases can reduce mental distress in response to a pandemic.”

*Reviewer: 2*

*The present study tested the effects of COVID-19 waves on mental health among medical staffs at 18 hospitals treating COVID-19 patients in Japan. Authors found that contrary to increasing new infected cases as waves progressed, initially elevated psychological distress (K6) and fear of COVID-19 (FCV-19S) were decreased among waves. These trends were more prominent in individuals with high trait anxiety and in occupations other than physicians or nurses.*

*This study is meaningful and interesting to examine longitudinal mental health data of medical healthcare workers for 21 months including COVID-19 pandemic waves. I suggest the following points to improve the manuscript.*

We appreciate the reviewer’s kind and careful review.

*1) Authors should state the response rate of the participants in each hospital to discuss sample bias.*

We thank the reviewer for giving us the opportunity to clarify this point. Information on the current survey was provided to candidates for participation via handouts and posters in each hospital. However, during the pandemic many medical staff left, started another job, or transferred to another department each month. For these reasons, it was difficult to accurately count the total number of candidates among the medical staff in each hospital each week. Therefore, in the current study an accurate response rate could not be calculated, nor could potential selection bias be completely eliminated. Considering this point, we have added the following sentences:

Methods:

Lines 97-99 of the revised manuscript: “In order to reduce selection bias, the staff in charge of this survey in each hospital widely called the survey to the attention of medical staff via handouts and posters that asked them to the questionnaire.”

Discussion:

Lines 403-408 of the revised manuscript: “Fourth, information on the current survey was provided to candidates for participation via handouts and posters in each hospital. However, during the pandemic many medical staff left, started another job, or transferred to another department each month. For these reasons, it was difficult to accurately count the total number of candidates among the medical staff in each hospital each week. Therefore, in the current study an accurate response rate could not be calculated, nor could potential selection bias be completely eliminated.”

*2) To evaluate effects of COVID-19 waves on mental health among medical staff, their experience of work in the COVID-19 unit or suffering from the illness are more important rather than their baseline anxiety and occupation. Why did authors not examine them?*

We thank the reviewer for giving us the opportunity to address this point. Unfortunately, we did not ask whether the participants had ever been infected with COVID-19. However, the data on direct contact with infected patients was available, so GLMMs were conducted with this data as the exposure. In these analyses, only participants who had direct contact with infected patients were included (n=451). The following sentences were added to the methods and results sections:

Methods:

Lines 136-137 of the revised manuscript: “In addition, participants were asked each time whether they had experienced direct contact with patients infected with COVID-19.”

Lines 154-156 of the revised manuscript: “Furthermore, the associations between direct contact with infected patients and psychological distress and fear of COVID-19 were examined in those participants who had direct contact with infected patients.”

Lines 168-171 of the revised manuscript: “Regarding the association between direct contact with infected patients and psychological distress and fear of COVID-19, whether there was direct contact or not was not fixed by the participant, but differed at the time of the response, so it was entered into the GLMM as a time-varying variable, without including an interaction term with time.”

Results:

Lines 278-281 of the revised manuscript: “As for direct contact with patients infected with COVID-19, 451 participants who had direct contact with infected patients were included. Such contact was not associated with psychological distress (B = .01, 95% CI: −.11 to .14), but was significantly associated with fear of COVID-19 (B = .72, 95% CI: .05 to 1.39).”

*3) I think data of K6 and FCV do not exhibit a normal distribution. Therefore, Generalized Linear Mixed Model may be more suitable than LMM for statistical analyses in such a case.*

We appreciate the reviewer’s excellent suggestion. According to the reviewer’s comment, in the revised manuscript we have changed the analytical method to the GLMM. The K6 scores did not exhibit a normal distribution, so we treated it as a negative binomial distribution; however, the FCV-19S scores could be assumed to have a normal distribution, so we treated it as a Gaussian distribution (please refer to the figures below). Changing the analysis method to GLMM did not conclusively change the results. The methods and results sections, as well as Table 2, were changed as follows:

Methods:

Lines 145-147 of the revised manuscript: “First, a generalized linear mixed model (GLMM) was used to examine longitudinal changes in psychological distress and fear of COVID-19 during the observation period; additionally, it was used to determine whether the amount of change differed according to individual attributes.”

Results:

Lines 231-232 of the revised manuscript: “For K6 scores (psychological distress), a GLMM that assumed a negative binomial distribution was performed because these scores were not normally distributed.”

Lines 259-260 of the revised manuscript: “For FCV-19S scores (fears of COVID-19), a GLMM assuming a Gaussian (normal) distribution was performed.”

Table 2

|  | Total observation period | |
| --- | --- | --- |
|  | Model 1 | Model 2 |
|  | B (95% CI) | B (95% CI) |
| Time (month) | -.02 (-.03, -.01)* | -.02 (-.07, .02) |
| Trait anxiety (below average group is reference) | | |
| High | .82 (.45, 1.19)* | 6.37 (-24.42, 37.16) |
| Very high | 1.55 (1.18, 1.91)* | 18.18 (-12.59, 48.94) |
| Time × Trait anxiety | | |
| High |  | -.01 (-.05, .03) |
| Very high |  | -.02 (-.06, .02) |
| Occupation type (other hospital staff is reference) | | |
| Doctor | -.26 (-.81, .30) | -17.26 (-50.42, 15.91) |
| Nurse | -.22 (-.52, .08) | -16.91 (-38.83, 5.00) |
| Time × Occupation type | | |
| Doctor |  | .02 (-.02, .07) |
| Nurse |  | .02 (-.01, .05) |
| Background characteristics | | |
| Age | -.001 (-.01, .01) | -.001 (-.01, .01) |
| Sex (female) | .52 (.15, .89)* | .55 (.18, .93)* |

*P <.05

*4) Cross correlation analysis make the central theme of the manuscript vague. Cross-correlation r values are relatively small. I wonder if these analyses are really necessary.*

We thank the reviewer for this insightful comment. We designed the current study to be exploratory in nature, and cross-correlation analysis was suitable for this purpose. We revised sentences taking both this point as well as Reviewer 1’s Comment 5 (on the revision of cross-correlation analyses) into consideration. Please see the response to Reviewer 1’s Comment 5.

*Reviewer: 3*

*The primary finding of this longitudinal study that anxiety/distress and fear of COVID increased early in the pandemic and then decreased with further exposure to the pandemic is worth presenting, but the report is much longer than is warranted by the finding. I would suggest greatly condensing all sections of the paper, focusing on the primary point.*

We have substantially revised the manuscript; in addition, some results were presented only in the supplemental materials in accordance with the reviewers’ suggestions.

*The suggestion about the duration of follow-up of staff may be less important than a recommendation about when and what kind of intervention might improve health care worker adaptation to pandemics.*

*You might consider as an additional explanation of your findings that physicians and nurses have more direct patient contact, so that repeated exposure to the stress of potential infection, especially without getting sick oneself, leads to tolerance to distress rather than intellectual mastery of it.*

We thank the reviewer for this insightful comment. Based on their suggestion, we added the following sentences:

Discussion:

Lines 362-364 of the revised manuscript: “It could also be expected that as physicians and nurses have more direct patient contact, repeated exposure to the stress of potential infection might lead to a greater distress tolerance, especially if they have not become ill themselves.”

*The impact of high trait anxiety could be a statistical as well as a psychological phenomenon.*

We thank the reviewer for this suggestion. In response to this comment, we added the following sentences:

Discussion:

Lines 411-414 of the revised manuscript: “Sixth, the group that scored very high on trait anxiety also had high psychological distress and fear of COVID-19 scores. However, this result could be a statistical phenomenon as well as a psychological phenomenon.”

*Finally, you might clarify whether you corrected statistically for multiple comparisons.*

We thank the reviewer for this comment. As suggested, we adopted the significance level after applying Bonferroni's correction for multiple comparisons.

Methods:

Line 194 of the revised manuscript: “Multiple comparisons were corrected using Bonferroni’s method.”

*Reviewer: 4*

*I read this manuscript with interest, however, the report is difficult to follow and several methodological issues exist:*

We appreciate the reviewer’s careful review and insightful comments.

*1. Some comments on the local COVID-19 situation and policies on the ground during the period the data was conducted would be helpful as well.*

We appreciate the reviewer’s comment. We added sentences about the local COVID-19 situation and policies in a paragraph in the method section as well as in the response to Comment 2 of Reviewer 1.

*2. Would the psychometric scores be high because of the generally high amount of uncertainty and fear during the early stages of the pandemic? How do we account for this?*

We thank the reviewer for pointing this out, and we agreed with the comment. Therefore, we added the following sentence:

Discussion:

Lines 328-329 of the revised manuscript: “It is possible that the psychometric scores were high because of the generally high amount of uncertainty and fear of COVID-19 during the early stages of the pandemic.”

*3. More details are necessary on the dissemination and sampling process. Did the authors take steps to guard against duplicate responses to the survey?*

We appreciate the reviewer for giving us an opportunity to clarify this important point. We added the following sentences:

Methods:

Lines 99-103 of the revised manuscript: “After the survey period, we checked all responses to make sure that the same individual did not answer more than once per day. If the hospital name, department name, and email account on two responses matched, we regarded them as having been completed by the same individual. If we found duplicate responses, we considered the last one to be the correct one and deleted the others.”

Discussion:

Lines 408-411 of the revised manuscript: “Fifth, it is possible that individuals changed their email addresses during the survey period, allowing for the assumption that another person had answered when in fact the same individual responded repeatedly. In this case, duplicate responses could have occurred and might have influenced the current results.”

*4. “The ethical committee of Hamamatsu University School of Medicine approved this study protocol as a retrospective study on 29 March 2022 and informed consent was obtained from participants in the form of an opportunity to opt out on the website (20-083)” - does this mean the study was approved retrospectively rather than prospectively? Unclear why this is the case. It seems the authors did not obtain the necessary ethical approval before embarking on the study, especially for a multi-site study.*

We appreciate the opportunity to clarify this point. During this pandemic, the mental distress of medical staff may have caused their resignation and led to subsequent overwhelming of hospitals in the prefecture. We performed a survey contracted by the prefecture to longitudinally investigate and monitor the degree of psychological burden experienced by medical staff. After finishing this survey, we obtained ethical approval and analyzed the results. Therefore, this study was designed as a retrospective one. To clarify this point, we revised the following sentences:

Methods:

Lines 82-86 of the revised manuscript: “The survey was contracted to us by the disability care division of the prefectural government, with the aim of longitudinally investigating and monitoring the psychological burden on medical staff. The survey was conducted from 1 February 2021 to 31 October 2022; this period covered the 3rd to 7th waves of the COVID-19 pandemic in Japan.”

Lines 103-105 of the revised manuscript: “After finishing the survey, we obtained ethical approval and analyzed the survey results. Therefore, this study was designed as a retrospective one.”

*5. “The current long-term longitudinal study” - redundant to say “long-term longitudinal”.*

We appreciate the reviewer’s suggestion. Based on the reviewer’s comment, we checked carefully and corrected similar redundancies throughout the manuscript.

*6. “… demographics at the basal assessment” - please change “basal” to “initial”.*

Once again, we thank the reviewer for this helpful suggestion. Based on the reviewer’s comment, we checked carefully and corrected similar cases throughout the manuscript.

*7. Under the occupational groups, health attendants and environmental services workers appear to be left out. This should be an area for future research as previous studies have found that they experience significant (if not greater) psychological stress than medical staff (citation: pubmed.ncbi.nlm.nih.gov/36116538).*

We appreciate the reviewer’s careful review and insight. Considering the suggested literature, we have added the following sentences:

Discussion:

Lines 414-417 of the revised manuscript: “Seventh, although health attendants and environmental services workers were not included in the current study, previous studies showed that they experience more significant psychological stress than medical staff [45]. Therefore, future studies should include them to further clarify the psychological differentiation among occupational groups.”

The suggested paper was added to the reference list:

Ng QX, Yau CE, Yaow CYL, Lim YL, Xin X, Thumboo J, et al. Impact of COVID-19 on environmental services workers in healthcare settings: a scoping review. J Hosp Infect. 2022;130: 95–103. doi:10.1016/j.jhin.2022.09.001

*8. Other study limitations were not discussed. In this panel survey study, it is hard to tell exactly who responded to the study and the individuals surveyed at the different time points may not be the same person.*

We appreciate the reviewer’s suggestion. We made some revisions in response to this comment as well as to Comment 3 of Reviewer 4.

*Also, what about the likelihood of selection bias (of unknown extent because attrition cannot be evaluated)?*

We thank the reviewer for this comment. Regarding potential selection biases, we revised sentences based on this comment as well as on Comment 1 of Reviewer 2.

*9. Given the economic impact of the pandemic, socioeconomic status may still vary over time amongst the individuals sampled, which was not assessed in the present study. This is a study limitation as well.*

We thank the reviewer for this insightful comment. In April 2023, the Japanese Nursing Association made public the survey result that 10.3% of new nurse graduates resigned within a year (<https://www.nurse.or.jp/english/>). However, the respective rates of resignation in other medical occupations were not reported; neither were those in the local study area. Additionally, a temporal allowance was provided to frontline doctors and nurses, and the wages of nurses and other medical staff in Japan were raised during the study period. However, exact information on the rates of increase in medical staff wages were not available. Considering the suggested points, we have added the following sentence:

Discussion:

Lines 417-419 of the revised manuscript: “Eighth, in the current study, the variation in socioeconomic status of medical staff during the pandemic was not considered because of a lack of sufficient data on resignation rates and wage changes during the study period.”

*10. “.. desirable period in which to follow up high-risk individuals after exposure to stressors (e.g. from 0 to 7 weeks)” - how was this derived? This is unclear.*

We thank the reviewer for the careful review of our manuscript. We have revised the sentence as follows:

Conclusions:

Lines 426-427 of the revised manuscript: “... exposure to stressors (e.g., from 0 to 7 weeks; based on the results of current cross-correlation analyses showing the timing of elevations of fear and anxiety).”
