# Supplementary material for: Time-course changes in mental distress and their predictors in response to the coronavirus disease 2019 (COVID-19) pandemic: A longitudinal multi-site study of hospital staff
Source: PLoS One. 2023 Oct 5;18(10):e0292302. doi: 10.1371/journal.pone.0292302 (PMC10553228; doi:10.1371/journal.pone.0292302)
Supplement: Attachment — Submitted filename: PONE-D-23-03982R2_response.docx [file pone.0292302.s007.docx]

21^st^ August 2023

Dr. Emily Chenette, PhD, and Dr. George Vousden, PhD

Editor-in-Chief, *PLOS ONE*

Dear Dr. Chenette and Dr. Vousden,

Thank you for your kind letter on 28^th^ July 2023, regarding our manuscript PONE-D-23-03982 entitled “**Time-course changes in mental distress and their predictors in response to the coronavirus disease 2019 (COVID-19) pandemic: A long-term longitudinal multi-site study of hospital staff.**” We greatly appreciate the comments and suggestions provided by the editor and reviewers for the re-revision of our manuscript.

We have substantially revised our manuscript in accordance with your suggestions, as well as those of the reviewers. Our point-by-point responses to all of the comments are presented below; reviewer comments are in italics with our responses immediately following each comment. The revised sections of the text are underlined in each response as well as in the revised manuscript itself. The line numbers in the responses were based on the marked up copy version of revised manuscript.

*Reviewer: 1*

*In this revision, the authors have done a good job responding to my initial concerns.*

We appreciate the reviewer’s kind and careful re-review.

*1) While reliability information is welcome in these two places, it could be made slightly more clear that these reliability coefficients come from other studies, not the current study. Also, it would be nice to get an additional remark about the validity of the K6 (viz., that it correlates with other measures or ratings of mental distress?), as was done for the FCV-19S.*

We thank the reviewer for giving us the opportunity to clarify this point. As suggested, the following sentences were added to the revised manuscript:

Methods:

Lines 114-118 of the revised manuscript: “A previous study reported that Cronbach’s α of the Japanese version of K6 scale was 0.85 [14]. The screening performance of this scale was comparable with that of Center for Epidemiologic Studies-Depression Scale (CES-D), and better than that of Depression and Suicide Screen (DSS) in the Japanese version [14].”

Lines 121-125 of the revised manuscript: “A previous study also revealed Cronbach’s α of the Japanese version of FCV-19S scale was 0.82 [9]. This scale showed a significant positive correlation to Generalized Anxiety Disorder (GAD)-7, Patient Health Questionnaire for Adolescents (PHQ-A), and Perceived Vulnerability to Disease Scale (PVDS) in the Japanese version. The scale had sufficiently high internal confidence and moderately good construct validity. [9]”

*2) Firstly, I remain unconvinced that the GLMM and cross-correlation models address the different measurement intervals problem. Once you've binned the data by week, the different measurement intervals are lost, right? Every participant's data are one week at a time, regardless of where in that week the observation(s) occurred. GLMM and DCCA are capable of addressing the variable intervals, but that problem is solved by binning the data by week. If the authors agree, then I'd suggest simply deleting the sentence, "However, the employed generalized linear mixed model allows for different measurement times and intervals for each individual, and detrended cross-correlation analysis is considered to be less influenced by these limitations."*

We thank the reviewer for this insightful comment. As suggested, the following sentences were deleted from the revised manuscript:

Discussion:

Lines 400-402 of the revised manuscript: "~~However, the employed generalized linear mixed model allows for different measurement times and intervals for each individual, and detrended cross-correlation analysis is considered to be less influenced by these limitations.~~"

*Second, the cross-correlation analyses remain unclear. In my review I requested the partial cross-correlation functions (PCCF), but then I referred to these as "PACF." In their reply, the authors have provided a partial autocorrelation function for each variable, presumably in response to the erroneous abbreviation in my third sentence, rather than the partial CROSS-correlation I referred to in my first sentence. I apologize for the inconsistency in my review, and I would now like to invite the authors to see whether the cross correlations they report would change by correcting them for autocorrelation within the variables. Conceptually, what I am asking them to look at is the extent to which autocorrelation accounts for the apparent lagged effects. The partial cross correlation corrects the lagged effects for the covariance accounted for by the concurrent and intervening effects. The authors have picked the peak in a smooth autocorrelation function to claim special status for, in the case of the K6, the 7 week lag. I just don't think this is supportable from these analyses. In short, the cross-correlation function needs to be interpreted in light of the partial cross-correlation function (PCCF) that corrects for autocorrelation and intervening effects.*

*That said, like Reviewer 2 (point #4), I do not view the lagged effects as crucial to the contribution of the manuscript, so if the authors end up electing to remove the cross correlation analyses and discussion entirely it would not alter my evaluation of the manuscript. The PCCF could, however, be quite interesting.*

We appreciate the reviewer’s insightful and important comments. We agreed with the comments and also thought that it would be rather important to focus on the time-course changes and the association between the outcomes and anxiety and occupation resulting from GLMM analysis than to emphasize the result of cross-correlation analysis. Therefore, we removed and revised the sentence and figures from the revised manuscript related to the result and discussion of cross-correlation analyses as follows.

Abstract:

Lines 33-36 of the revised manuscript: ”~~Cross-correlation analyses demonstrated that K6 and FCV-19S exhibited time-course changes, with increases in these indices occurring simultaneously with an increase in infections and decreases delayed by 7 weeks after an increase in infections.~~”

Lines 38-39 of the revised manuscript: “~~The results also indicated a desirable period for following up high-risk individuals after exposure to stressors.~~”

Introduction:

Lines 62-66 of the revised manuscript: “ ~~Furthermore, high temporal resolution is necessary to test the relationships between mental distress, such as fear or anxiety, and the onset of stressors, because previous studies have indicated that these relationships operate at the scale of days to weeks [8]. However, the temporal resolution of most previous longitudinal research on COVID-19 mental health has been limited to months to years, with a limited number of time-points [9].~~”

Lines 74-76 of the revised manuscript: ”~~Additionally, using a high temporal resolution analysis of long-term longitudinal data, the time-course changes in the relationship between the pandemic waves and mental distress were also examined.~~“

Methods:

Lines 184-194 of the revised manuscript: “~~Next, to examine how the number of newly infected cases of COVID-19 was correlated with psychological distress and fear of the COVID-19, respectively, we conducted cross-correlation analyses. Cross-correlation analysis allows us to understand the similarity of data in a time series and the lag of the period. The cross-correlation between the number of newly infected cases in the prefecture and the scores for psychological distress / fear of COVID-19 were all calculated as weekly averages. We utilized weekly averages in the analyses, because mental health surveys were not completed by each participant on each day; therefore, when the unit of time was set to one day, the amount of missing data was very large. Furthermore, because the number of newly infected cases tended to increase, and because psychological distress and fear of COVID-19 could also have a trend through the period, we conducted a detrended cross-correlation analysis (DCCA) using standardized values.~~”

Lines 195-196 of the revised manuscript: “Additionally, the observation period was divided into two periods (before and after 100 weeks), and a GLMM ~~and DCCA~~ was then conducted for each period.”

Lines 200-201 of the revised manuscript: “GLMM was conducted using STATA version 17.0 [22]. ~~and DCCA was conducted using Python version 3.6.~~

Result:

Lines 286-311 of the revised manuscript: we removed the section of the result of cross-correlation analysis.

Discussion:

Lines 323-327 of the revised manuscript: “~~Focusing on each pandemic wave, psychological distress and fear of COVID-19 also showed time-course changes as waves with increases that were delayed by 0–2 weeks after an increase in the number of infections and decreases that were delayed by 7 weeks after an increase in the number of infections. The wave of fear regarding COVID-19 tended to be slightly delayed after the wave of psychological distress.~~”

Lines 378-391 of the revised manuscript: “~~The present study showed time-course changes in psychological distress and the fear of COVID-19 with increases in the distress and fear delayed by 0–2 weeks after an increase in the number of infections and decreases in the distress and fear delayed by 7 weeks after an increase in the number of infections. Previous research on the effects of acute stressors on anxiety and psychological reactions in various populations have shown responses to stressors within a few weeks [43]. Previous longitudinal studies with weekly measurement under the COVID-19 pandemic were limited. A large-scale 20-week longitudinal study from March to August 2020 reported that anxiety and depression levels both declined across the first 20 weeks following the introduction of lockdown in England, suggesting an adaptation for the situation. The fastest decreases were observed across the strict lockdown period (i.e., 2 to 5 weeks), with symptoms plateauing as further lockdown easing measures were shown (16 to 20 weeks) [44]. The present results across pandemic waves revealed the peak and subsequent decrease of distress and fear after a stressor extended current knowledge regarding the desirable timeframe for mental health care under the onset of stressors such as a pandemic of infectious disease, as well as other disasters.~~”

Lines 396-402 of the revised manuscript: “~~To reduce the potential confounding effect, weekly averages were calculated to summarize the observations. These weekly averages could make the temporal resolution insufficient, as the lag between the peak of increase in the number of infections and the peaks of psychological distress and fear of COVID-19 could have occurred at intervals shorter than one week. However, the employed generalized linear mixed model allows for different measurement times and intervals for each individual, and detrended cross-correlation analysis is considered to be less influenced by these limitations~~.”

Conclusion:

Lines 429-431 of the revised manuscript: “~~Furthermore, the current study suggests a desirable period in which to follow up high-risk individuals after exposure to stressors (e.g., from 0 to 7 weeks; based on the results of current cross-correlation analyses showing the timing of elevations of fear and anxiety).~~”

Supporting information:

Lines 608-614 of the revised manuscript: “~~S2 Fig. Cross-correlation between weekly average number of newly infected COVID-19 cases and K6 score for (A) 100 weeks† or earlier and (B) after 100 weeks. †The day of the COVID-19 outbreak in Japan is defined as “week 1” (January 16, 2020).~~

~~S3 Fig. Cross-correlation between weekly average number of newly infected COVID-19 cases and FCV-19S score for (A) 100† weeks or earlier and (B) after 100 weeks. †The day of the COVID-19 outbreak in Japan is defined as “week 1” (January 16, 2020).~~”

Figures:

We removed Fig 3, S2 Fig, and S3 Fig.

*Reviewer: 3*

*You have answered many of the issues raised by reviewers, but the manuscript is still very long considering the information provided.*

We appreciate the reviewer’s helpful comment. Together with the suggestions by Reviewer 1, we removed all the sentences and figures related to cross-correlation analysis, and these revision made the manuscript more concise. The main text of re-revised manuscript is 4,003words, while that of revised manuscript was 4,883 words.

*The recommendation that as staff encounter more cases they will become desensitized if they do not become ill themselves is likely to be correct, but you still might add recommendations for specific interventions, or state that no intervention is necessary.*

Thank you for giving us this opportunity to further clarify the issue. e have revised the text regarding the intervention as follows:

Line 360-368 of the revised manuscript: “Doctors and nurses experienced less psychological distress and fear of COVID-19 than individuals in other hospital occupations. It could also be expected that as physicians and nurses have more direct patient contact, repeated exposure to the stress of potential infection might lead to a greater distress tolerance, especially if they have not become ill themselves. This could also be attributed to their comparably easier access to medical information about COVID-19 or their greater ability to take precautions against infection than other hospital workers. Therefore, taken together, the present results support the possibility that some intervention for increasing knowledge and access to medical information about infectious diseases and taking precautions can reduce mental distress in response to a pandemic.”

In addition, we also carefully check the current manuscript and revised the sentences as follows:

In the abstract, the statistical results were added for the FCV-19S as follows:

Lines 28-31 of the revised manuscript: “Contrary to increasing new infected cases as waves progressed, initially elevated psychological distress (K6) and fear of COVID-19 (FCV-19S) were decreased among waves (K6: B = -.02, 95% confidence interval [CI] = -.03 to -.01; FCV-19S: B = -.10, 95% CI = -.16 to -.04).”

In the Table 1, the layout was corrected and “K6” was added as follows:

Row 2: “Outcome measures at the initial assessment ~~545 6.01 5.29 0 24~~”

Row 3: “K6 545 6.01 5.29 0 24”

In the Supporting information, the title of supplemental tables were added as follows:

S1 Table: “S1 Table. Effects of time and trait anxiety or occupation type on K6 scores for 100 weeks or earlier and after 100 weeks”

S2 Table: “S2 Table. Effects of time and trait anxiety or occupation type on FCV-19S scores for 100 weeks or earlier and after 100 weeks”
